# Volume-Staged Radiosurgery for Large Arteriovenous Malformation: Retrospective Analysis of 19 Cases

**DOI:** 10.7759/cureus.16901

**Published:** 2021-08-05

**Authors:** Takashi Shuto, Shigeo Matsunaga

**Affiliations:** 1 Department of Neurosurgery, Yokohama Rosai Hospital, Yokohama, JPN

**Keywords:** arteriovenous malformation, volume-staged radiosurgery, gamma knife surgery, radiosurgery, large volume

## Abstract

Introduction

The effectiveness of Gamma Knife surgery (GKS) for small arteriovenous malformations (AVMs) is well known. However, for large AVMs, the prescribed dose should be decreased to reduce the risk of radiation damage, but it leads to a decrease in nidus obliteration rates. Therefore, it is very difficult to achieve complete obliteration of large AVMs in a single treatment, and methods using multiple irradiation such as volume-staged stereotactic radiosurgery (VS-SRS) have been suggested. We retrospectively reviewed our results of VS-SRS for large AVMs to assess the efficacy of VS-SRS.

Methods

Nineteen patients with AVMs of ≥10 ml and who consented to VS-SRS were treated by this surgical strategy and retrospectively analyzed. We excluded AVMs that were too large such as those >40 cc to avoid severe radiation damage. The components were divided mainly in the vertical direction, and each component was irradiated with a marginal dose of 18 Gy. Each irradiation was performed at intervals of 3-6 months, and the components with main feeders were irradiated first, and the components that included the main drainer were irradiated last. We tried to keep V18 to <10 ml if possible. The follow-up after GKS was performed by MRI every 6 months, and cerebral angiography was performed to confirm complete nidus obliteration, but if the patient refused, it was judged on the basis of MRI findings.

Results

Nineteen patients with a mean age of 40.2 years underwent VS-SRS. Each compartment was irradiated at 3­-16 month (median, 3 months) intervals. The mean initial AVM volume was 19 ± 5.6 ml. Fourteen patients received two-stage radiosurgery and five received three-stage radiosurgery. The median target volume was 9.1 ml at stage 1, 9.0 ml at stage 2, and 10.1 ml at stage 3. The median margin dose was 18 Gy at each stage. The mean follow-up after the last stage of radiosurgery was 3.9 (1-11.4) years. Complete obliteration was confirmed by angiography in six patients, and by magnetic resonance angiography in one patient. The cumulative obliteration rates were 30.7% and 58.2% at 3 and 5 years following VS-SRS, respectively. The cumulative hemorrhage rates were 7.1% and 22.1% at 3 and 5 years, respectively. MRI showed T2-weighted prolongation in 15 patients (78.9%). Of these 15 patients, four were symptomatic (epilepsy in all) and two underwent surgical removal of symptomatic expanding hematomas.

Conclusions

In our experience, VS-SRS offers a viable treatment strategy in patients with large AVMs. Further optimization of the dose and volume at each stage is required.

## Introduction

The effectiveness of Gamma Knife® (Elekta, Instrument AB Stockholm, Stockholm, Sweden) surgery (GKS) for relatively small arteriovenous malformations (AVMs) is widely known [1]. According to a multicenter study of 2,236 patients by Starke et al., an average of 4.3 ml of the nidus was irradiated with an average marginal dose of 20.5 Gy and a 7-year mean follow-up finally showed that complete obliteration was achieved in 64.7% of the patients [1]. However, for large AVMs, the prescribed dose should be decreased to reduce the risk of radiation damage, but it leads to a decrease in nidus obliteration rates. Therefore, it is very difficult to achieve complete obliteration of large AVMs in a single treatment, and methods using multiple irradiation have been reported previously [2-9].

For relatively large AVMs such as those >10 ml, we divide the nidus into multiple components mainly along the z-axis and irradiate them step by step volume-staged stereotactic radiosurgery (VS-SRS). Although VS-SRS is a classical approach for large AVMs [2,3,5-9], the optimal dose, irradiated volume, and appropriate interval remain unclear. The study aimed to evaluate our results of VS-SRS for large AVMs >10 ml.

## Materials and methods

Patients with AVMs of ≥10 ml and who consented to VS-SRS were treated by VS-SRS strategy. We excluded AVMs that were too large such as those >40 ml to avoid severe radiation damage. We retrospectively analyzed 19 patients with large AVMs (14 males, five females; average age, 40.2 years) who underwent VS-SRS in our hospital (Tables 1, 2). Onsets were bleeding in eight patients, convulsions in six, and others in five. Treatment outcomes are shown in Table 3.

**Table 1:** Characteristics of patients M: male; F: female; CF: cyst formation; CO: complete obliteration; UO: under observation; EH: expanding hematoma

Case	Age/sex	Onset	Interval (months)	Total volume (ml)	1st dose (Gy)	1st volume (ml)	2nd dose (Gy)	2nd volume (ml)	3rd dose (Gy)	3rd volume (ml)	Follow-up (years)	Clinical complication	Obliteration
1	45M	Epilepsy	7	15.4	18	7.5	18	7.9	－	－	11.4	EH	CO
2	19M	Headache	4	15.3	18	8.3	18	7	－	－	10.4	EH	－
3	28M	Epilepsy	3	20.1	18	7	18	13.1	－	－	8.3	Hemorrhage	－
4	19M	Epilepsy	16	15.8	18	8.1	18	7.7	－	－	6.3	－	UO
5	43F	Hemorrhage	3	24.5	18	7.3	18	11.2	18	6	2.7	－	CO
6	50F	Hemorrhage	4	30.9	18	9.8	18	9.1	18	11.9	2.4	－	UO
7	45M	Incidental	3	16.4	18	8.9	18	10	－	－	2.3	－	CO
8	38M	Incidental	4	14.1	18	8.5	18	5.6	－	－	1.8	－	CO
9	42F	Hemorrhage	6	26.1	18	8.1	18	9	18	9.1	1.0	Hemorrhage	－
10	50M	Epilepsy	3	26	18	7.8	18	8	18	10.2	2.8	－	UO
11	46M	Incidental	4	17	18	7.6	18	9.4	－	－	1.7	－	CO
12	39M	Hemorrhage	3	10.9	18	6.4	18	4.5	－	－	5.3	CF	CO,
13	46F	Hemorrhage	3	13.9	18	8	18	5.9	－	－	3.2	－	CO
14	34M	Epilepsy	3	19.4	18	10.9	18	8.5	－	－	3.0	－	UO
15	33F	Headache	3	54	18	15.6	18	13.9	18	13.4	1.8	－	UO
16	70M	Hemorrhage	3	23	18	10.7	18	12.5	－	－	2.8	－	UO
17	20M	Hemorrhage	3	18	18	10.3	18	7.1	－	－	2.0	Hemorrhage	UO
18	50M	Hemorrhage	4	34	18	18.4	18	15.9	－	－	2.8	－	UO
19	47M	Epilepsy	3	10.5	18	6.1	18	4.4	－	－	1.7	－	UO

**Table 2:** Summary of patients

Gender (M/F)	14/5
Mean age, years (range)	40.2 (19–70)
Onset	
Hemorrhage	8
Epilepsy	6
Others	5
Prior embolization	11
Mean total volume, ml (range)	24.8 (10.5–54)
Spetzler-Martin grade	
I	0
II	0
III	8
IV	8
V	3
Mean follow-up, years (range)	3.9 (1–11.4)

**Table 3:** Summary of treatment

Two-stage radiosurgery	14 patients
Three-stage radiosurgery	5 patients
Median time between stages, months (range)	3 (3–16)
Median volume per stage, ml (range)	8.3 (4.4–18.4)
Median margin dose per stage, Gy (range)	18 (all)

Preradiosurgical embolization was performed in 11 patients, and most procedures were performed by a referral neurosurgeon. The main purpose of pre-SRS embolization was occlusion of bleeding point to reduce the risk of rebleeding or volume reduction of nidus. Patients were followed up at our hospital following embolization.

The basic treatment strategy is summarized as follows:

1. The components were divided mainly in the vertical direction (z-axis direction), and each component was irradiated with a marginal dose of 18 Gy, which was planned so that the irradiation volume of 18 Gy (V18) did not exceed approximately 10 ml if possible.

2. Each irradiation was performed at intervals of 3-6 months, and the components with many feeders were irradiated first, and the components that included the main drainer were irradiated last.

3. Irradiation was postponed if the T2 hyperintensity due to radiation injury was prominent at the second or third irradiation.

At the first stage, after fixing the Leksell frame (Elekta AB, Stockholm, Sweden) under local anesthesia, the treatment was planned on the basis of MRI (gadolinium-enhanced time-of-flight images and proton density-weighted images), thin-slice contrast-enhanced CT, and cerebral angiography. Usually, cerebral angiography was performed only for the first stage. In the first treatment, we tried to irradiate the components with main feeders and kept V18 to <10 ml if possible.

The follow-up after GKS was performed by MRI every 6 months, and cerebral angiography was performed to confirm complete nidus obliteration, but if the patient refused, it was judged on the basis of MRI findings.

## Results

Fourteen patients received two-stage and five patients received three-stage radiosurgery. The average volume of the first irradiation was 9.1 ml, the second was 9.0 ml, and the third was 10.1 ml. Each compartment was irradiated at 3- to 16-month (median, 3 months) intervals. In most cases, the interval was 3 or 6 months except for one (Case 4, 16 months) who developed radiation-induced cerebral edema after the first stage procedure.

A summary of the treatment results is shown in Table 4.

**Table 4:** Summary of treatment results

Mean follow-up, years (range)	3.9 (1–11.4)
Treatment results (There is some overlapping)	
Complete obliteration (angiographic obliteration)	7(6)
Hemorrhage (death)	3 (1)
Expanding hematoma	2
Cyst formation	1
Under observation	8
Needed direct surgery	4

The average follow-up period after the final irradiation was 3.9 (1-11.4) years. Complete obliteration was achieved in seven patients, including confirmation by magnetic resonance angiography (MRA) in one patient, bleeding occurred in four patients, and surgery was necessary in four patients (two for expanding hematoma and two for hemorrhage). Seven patients continued follow-up before confirmation of complete occlusion, but in all seven, MRA showed a reduction of the nidus, suggesting that the obliteration mechanism was progressing.

**Figure 1:**
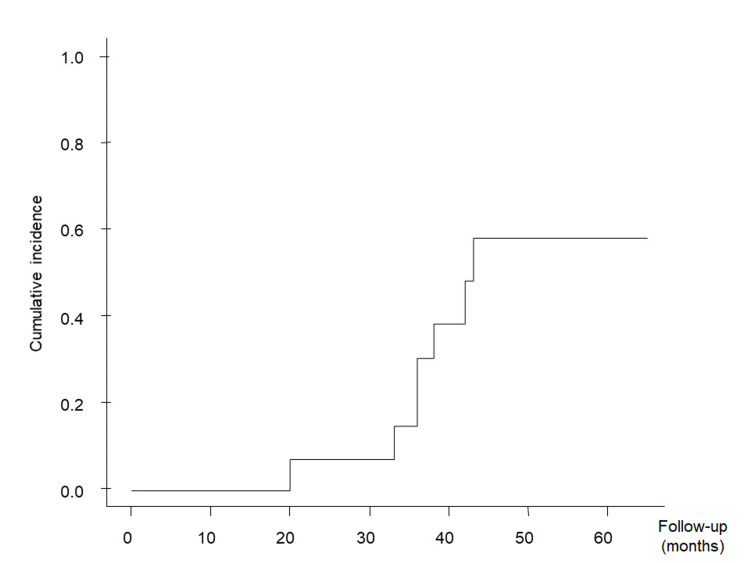
Cumulative obliteration rates Kaplan-Meier plot showing cumulative obliteration rates following volume-staged radiosurgery.

**Figure 2:**
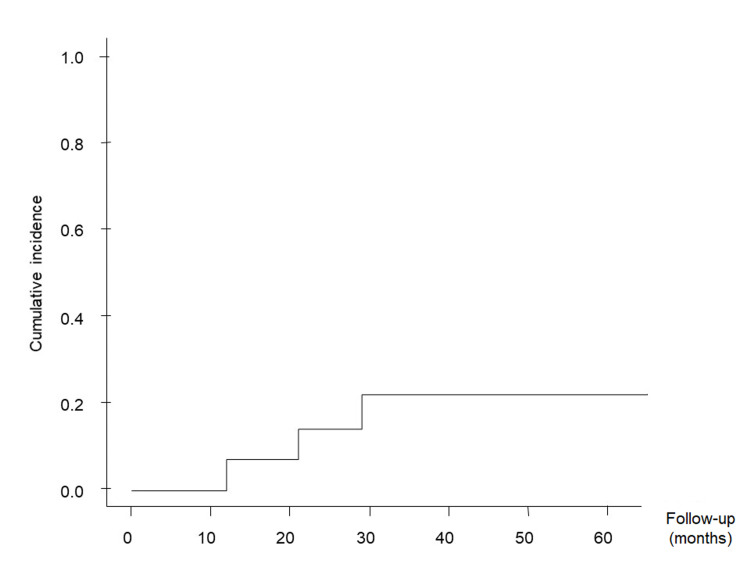
Cumulative hemorrhage rates Kaplan-Meier plot showing cumulative hemorrhage rates following volume-staged radiosurgery.

The cumulative obliteration rate according to the competing risk analysis was 30.7% at 3 years and 58.2% at 5 years (Figure 1). The cumulative bleeding rate was 7.1% at 1 year, 22.1% at 3 years, and 22.1% at 5 years (Figure 2).

Post-irradiation high-intensity signals on T2-weighted imaging of the nidus were observed in 15 (78.9%) patients, four of whom were symptomatic, and all had convulsions but none were persistent.

**Figure 3:**
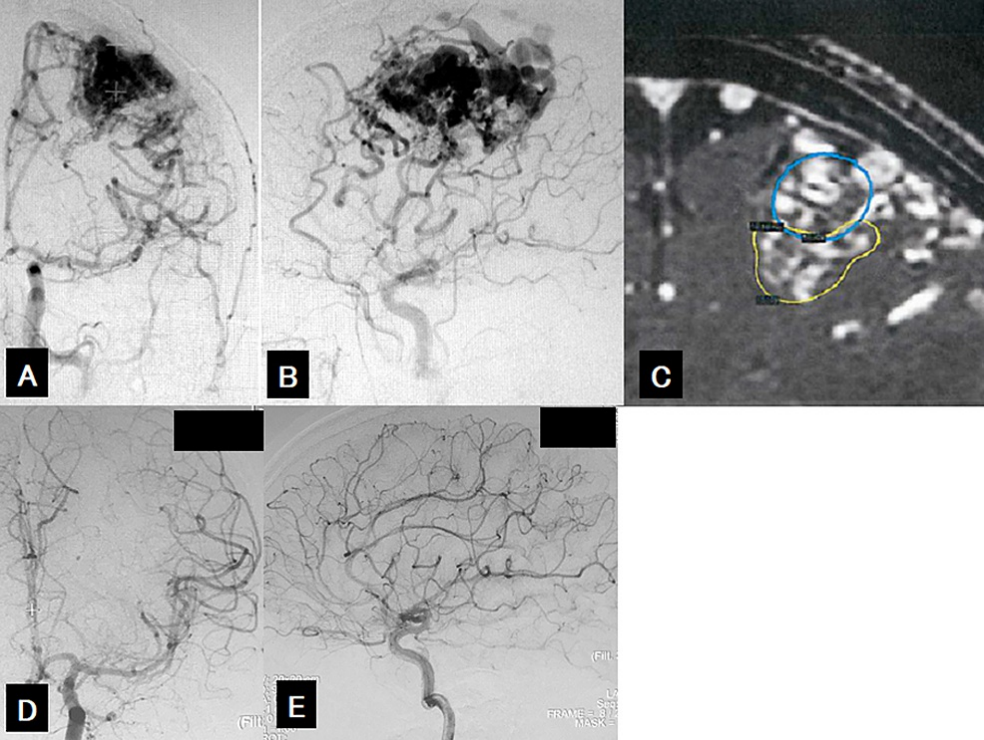
Illustrative case (Case 7) A 45-year-old man was diagnosed as having a cerebral AVM in the left frontal lobe during a medical checkup and was referred to our hospital for GKS following two embolizations at another hospital. Angiograms show a large arteriovenous malformation located at the left frontal and parietal lobe (A: anteroposterior view, B: lateral view). Given the large nidus volume, VS-SRS was performed by dividing the nidus into two parts. Radiosurgical plan shows the anatomical components of stage 1 (C: blue line, 8.9 ml) and stage 2 (C: yellow line, 10 ml). Cerebral angiography obtained 33 months after second radiosurgery shows complete obliteration of the nidus (D: anteroposterior view, E: lateral view). AVM: arteriovenous malformations; GKS: Gamma Knife surgery; VS-SRS: volume-staged stereotactic radiosurgery

Two patients developed expanding hematomas that were surgically removed, but no neurological deficits persisted. Bleeding occurred in four patients (Cases 3, 6, 9, 17), at 80, 29, 12, and 24 months, respectively, after the last irradiation. In all four patients, complete obliteration had not been confirmed. One patient died (Case 6), and one underwent craniotomy for hematoma removal (Case 3) but severe disability persisted. The other two patients were treated conservatively for bleeding: one with higher brain dysfunction (Case 9), and the other with no neurological sequelae (Case 17). An illustrative case (Case 7) is shown in Figure 3.

## Discussion

In this study, we found that VS-SRS for large AVMs was effective. For relatively large AVMs >10 ml, two main treatment strategies have been suggested: One is a method in which the entire nidus is irradiated with a low dose, such as 14-16 Gy, and additional irradiation is considered after observing changes after several years (dose-staged radiosurgery: DS-SRS) [4]. Another method is VS-SRS, which divides a large nidus into several compartments and prescribes a reasonable dose to each compartment at intervals [2,3,5-9]. Ilyas et al. conducted a systematic review of VS-SRS and DS-SRS and reported that the average complete obliteration rate was 41.2% for VS-SRS and 32.3% for DS-SRS [10]. Symptomatic radiation-induced changes, post-irradiation bleeding, and death were, respectively, 13.7%, 19.5%, and 7.4% for VS-SRS and 12.2%, 10.6%, and 4.6% for SDS-SRS. VS-SRS is advantageous from the standpoint of obtaining complete obliteration, but DS-SRS has fewer adverse events. Empirically, the degree of response to low-dose irradiation with DS-SRS varies greatly from patient to patient, and for this reason, we selected VS-SRS since 2009. Since the dose distribution of the GK is particularly steep in the z-axis direction, the compartments are mainly divided in the vertical direction (z-axis direction) to minimize the overlap of irradiation fields. The irradiation interval is approximately 4 months, but if cerebral edema due to radiation injury becomes evident, the irradiation is postponed, and the second or third irradiation is performed after improvement is shown on imaging findings.

As presented in the Results section, the cumulative obliteration rate in our series was by no means a satisfactory result; however, given that large AVMs are difficult to treat using other means, these results are likely the best that we can achieve at present.

Pollock et al. reported the treatment results of 34 patients who underwent VS-SRS between 1997 and 2012 [6]. The median stage was 2, the median volume of nidus was 22.2 ml, and the marginal dose was approximately 16 Gy. At a median follow-up of 8.2 years, complete obliteration was achieved in 53% of the patients. The cumulative total obliteration rate was 14% at 3 years, 54% at 5 years, and 75% at 7 years, and the cumulative total obliteration rate at 5 years was similar to our result. Bleeding after VS-SRS occurred in 18% of patients, and the cumulative bleeding rate after VS-SRS was 6% for 1 year, 12% for 3 years, and 19% for 7 years.

In VS-SRS, since a relatively large volume is irradiated multiple times, there is a strong concern about radiation damage. From our study, the incidence of transient cerebral edema after VS-SRS appeared to be high (15 of 19 patients, 78.9%). In our series, approximately 25% of the patients who had radiation-induced edema became symptomatic, and all had convulsions, but none developed permanent disabilities. Expanding hematomas were observed in two patients, but it was surgically removed easily without neurological sequelae.

To further assess the treatment effect and reduction of the complication risk in VS-SRS, the desirable irradiation volume, marginal dose, and irradiation interval at each stage will be studied in the future.

Seymour et al. analyzed the records of 69 patients who underwent VS-SRS performed from 1992 to 2008 that were divided into two groups: 1992 to 2004 and 2004 to 2008 [7]. In 2004-2008 group, the irradiation volume of each stage was reduced (median, 15 ml to 6.8 ml), the dose was increased (median, 15.5 Gy to 17 Gy), and the irradiation interval was shortened (median, 5.8 months to 3.7 months) relative to those of the treatment group 1992-2004. Multivariate analysis showed that the factors significantly associated with obliteration were the dose at each stage, compact nidus, and the volume of the entire AVM. The complication rate was 29% in the 1992-2004 group and 13% in the 2004-2008 group. Based on these findings, Seymour et al. recommended that the irradiation volume of each stage should be ≤8 ml and that the marginal dose should be ≥17 Gy [7].

In the future, it is anticipated that the volume, dose, and irradiation interval for each stage will be optimized after the accumulation of more cases.

In conclusion, VS-SRS by GKS can be considered as a treatment strategy for relatively large AVMs >10 ml. However, the risk that direct surgery due to bleeding or radiation damage will be required is not low. The volume and dose at each VS-SRS treatment stage requires further optimization.

## Conclusions

VS-SRS by GKS can be considered as a treatment strategy for relatively large AVMs >10 ml. Symptomatic radiation-induced edema is not uncommon, but permanent disabilities is rare. However, bleeding or a late complication such as expanding hematoma can occur. The volume and dose at each VS-SRS treatment stage requires further optimization.
